# Bullying and Incivility Experiences of Undergraduate Orthoptic Students on Clinical Placement

**DOI:** 10.22599/bioj.368

**Published:** 2025-03-21

**Authors:** Konstandina Koklanis, Meri Vukicevic, Andrea Simpson, Bojana Šarkić

**Affiliations:** 1 La Trobe University, AU; 2 Charles Darwin University, AU

**Keywords:** bullying, incivility, orthoptics, students, clinical placements

## Abstract

**Introduction:**

Clinical placements in allied health are crucial for students to develop skills in real-world settings. However, these environments can expose students to incidents of incivility, bullying, or harassment. Whilst much research has explored bullying in medicine and nursing, little is known about the rate or effect of bullying in smaller allied health professions. This study aimed to investigate the frequency of bullying incidents among final year orthoptic students and assess the consequential effects of this experience.

**Methods:**

In this cross-sectional study, final year orthoptic students and graduates who had completed placements in the preceding year were invited to complete an online survey. The survey instrument was adapted from the Clinical Workplace Learning Negative Acts Questionnaire-Revised. Information on demographics, placement attributes, bullying experiences, and their effects was gathered. The data were analysed using descriptive statistics.

**Results:**

A total of 20 individuals responded to the survey; 12 (60%) final year students and 8 (40%) graduates. Almost all participants (95%) reported experiencing at least one negative act whilst on placement, with 10 (50%) indicating they experienced bullying. Of these 10, all reported feeling humiliated by the incidents, and 90% reported a loss of confidence. Almost all students (90%) did not report the behaviour when it happened, with most students also being unaware of bullying and harassment policies of the university or health facility.

**Conclusion:**

Bullying and incivility pose challenges for orthoptic students during placements. This study highlights gaps in policy implementation and underscores the need for effective measures to address this issue.

## Introduction

Undergraduate education in allied health generally encompasses both theoretical instruction and hands-on practice, which includes work integrated learning where students undertake clinical placements or internships. Bullying, harassment, and incivility within workplace settings where clinical education takes place have been identified as a significant issue within the literature (Graj et al., 2019; MacMillan et al., 2022). However, much of the research to date has focused on nursing (Birks et al., 2018; Graj et al., 2019; Minton and Birks, 2019) and medicine (Clements et al., 2020; Colenbrander et al., 2020; Henning et al., 2023) with limited research in allied health professions. Within allied health, the focus has primarily been on physiotherapy students (MacMillan et al., 2022; Seager, 2004; Whiteside et al., 2014), and no research has explored bullying within smaller disciplines such as orthoptics.

Bullying is a term that is generally characterised by the persistent exposure to negative acts that can lead to significant harm (Einarsen et al., 2009). Where the behaviour is isolated, it may be referred to as incivility (Einarsen et al., 2009); however, often in the literature, bullying and incivility are used interchangeably (Courtney-Pratt et al., 2018). In general, bullying may take the shape of verbal, psychological, physical, and social attacks, and this often occurs where there is a power disparity. Within the healthcare setting, students find themselves in an environment with a dual purpose (Smith-Han et al., 2020); a workplace where clinical services are provided to patients and a learning environment where there is a power imbalance between staff and students. This can invariably lead to tension, particularly where there are increasing demands on clinical staff (Budden et al., 2017). The consequences of the negative behaviours within the healthcare education are far-reaching, affecting not only the well-being of students, but also the overall quality of care provided to patients (Layne et al., 2019). Students who experience bullying report diminished self-esteem, increased anxiety, and a decline in academic performance (Lund and Ross, 2017). Moreover, they often grapple with a persistent sense of unease, feelings of apprehension, and doubts about their abilities and career path (Budden et al., 2017; Courtney-Pratt et al., 2018). This can consequently lead to a student being unwilling to ask questions, a loss of motivation or commitment to patients, and an increased risk of making clinical errors (Ekici and Beder, 2014).

Understanding the encounters of students exposed to bullying during clinical placements holds great importance, as it directly influences student learning and wellbeing, in addition to the care patients may receive. Whist research has been undertaken in various allied health disciplines, no research to date has specifically explored the experiences of orthoptic students on placement as it relates to bullying. The aim of this study was to investigate the rate of incivility and bullying experienced by undergraduate orthoptic students during their final year of clinical placements and to explore the impact and responses to bullying.

## Methodology

### Study design

This was cross-sectional study that included the distribution of an online survey at two-time points; August 2021 and August 2022. The study adhered to ethical guidelines as outlined in the National Statement on Ethical Conduct in Human Research (2018) and received approval from the La Trobe University Human Research Ethics Committee (Ethics ID: HEC21096).

### Participants

Final year students of the La Trobe University undergraduate orthoptic program and graduates who had completed their orthoptic studies in the preceding year were invited to participate. Final year students were selected as this cohort undertakes block placements, which includes the highest volume of placement days. The study was advertised via email to current students and via the professional association Orthoptics Australia to new graduates. A total of 106 students were invited to complete the survey.

### Survey

The survey distributed was an adaptation of the Clinical Workplace Learning Negative Acts Questionnaire-Revised (CWL NAQ-R) (Smith-Han et al., 2020). The CWL NAQ-R is divided into two parts. Part (a) includes demographic questions and Part (b) consists of the 31 validated items that measure experiences of bullying, harassment, and incivility. For the CWL NAQ-R scale, participants are asked to rate how often they experienced specific behaviours during their clinical placements or internship using a 5-point Likert scale, which ranges from one to five; 1 = Never, 2 = Now and then, 3 = Monthly, 4 = Weekly, and 5 = Daily.

In our study, Part (a) included 11 questions which were adapted from the CWL NAQ-R to reflect the Australian context and incorporated new questions regarding placement characteristics and student awareness of bullying policies. Part (b) reflected the validated CWL NAQ-R scale and participants were asked to consider the clinical placements they had undertaken in the last 12 months. For final year students, the placements were completed in the last 6 months, and for graduates, the placements were completed in the preceding year. A third section, Part (c) was also added to the survey to collect further information regarding bullying, as suggested by Smith-Han et al. (2020), who designed the CWL NAQ-R. For Part (c), a definition of bullying was provided as follows: “Bullying takes place when one or more persons systematically and over time feel that they have been subjected to negative treatment on the part of one or more persons, in a situation in which the person(s) exposed to the treatment having difficulty in defending themselves against them It is not bullying when two equally strong opponents are in conflict with each other” (Einarsen et al., 1996 p. 20). Participants were asked to reflect on whether their responses to the CWL NAQ-R scale items could be considered as bullying based on this definition. Where participants responded positively, they were directed to 11 questions regarding the perpetrator and response to or the effect of bullying. Where participants responded negatively, the survey concluded. All questions in the survey were forced choice, with 9 questions within Part (a) and 10 questions within Part (c) including an ‘other’ option. Respondents who selected ‘other’ were able to provide free text answers to specify.

Prior to distribution, the adapted survey underwent a pilot test with six university colleagues and students, leading to minor revisions to enhance clarity and user-friendliness of Parts (a) and (c). Appendix 1 details the questions included in the survey.

### Procedure

Invited participants were directed to the online survey link via email. The survey was administered using Research Electronic Data Capture (REDCap) and commenced with an electronic participant information statement and request for consent. Only those who provided informed consent proceeded to complete the survey.

### Data analysis

Data was exported from REDCap and imported into SPSS version 27 for analysis (SPSS Inc., Chicago, IL, USA). Descriptive statistics, including frequencies and percentages, were performed. Free text responses were analysed using content analysis.

## Results

### Part A: Participant and clinic characteristics

#### Participants

The study included 20 participants,14 females and 6 males. This represents a response rate of 18.9%. Of the 20 participants, 12 (60%) were final year students, and 8 (40%) were graduates who had completed placements in the preceding year. 17 (85%) were aged 20–30, 3 (1%) were aged between 31–40, and 1 (5%) was aged between 41–50.

English served as the primary language for a vast majority of the participants (n = 17; 85%), with 11 (55%) reporting using a language other than English at home. These languages included Italian, Greek, Cantonese, Mandarin, Nepali, Persian, Serbian, and Turkish. No students identified as Aboriginal and/or Torres Strait Islander.

#### Clinical placement characteristics

The participants’ clinical placements were primarily in public hospital outpatient eye clinics (100%, n = 20) and private eye clinic (80%, n = 16) Geographically, clinics were distributed across metropolitan (100%, n = 20), regional (50%, n = 10), and remote (5%, n = 1) locations.

#### Bullying and harassment policies

Of the 20 participants, 6 (30%) indicated that there were not aware of the university policy on bullying and 8 (40%) reported that they could not recall if they had learnt about the policy. With regards to the bullying and harassment policies of the healthcare facilities where placements were undertaken, 12 (60%) participants reported that they were not aware of the related policies and 1 (25%) reported they could not recall. When asked if information or training was provided regarding what to do if bullying was experienced, 18 (90%) indicated they had not received information or training.

### Part B: Clinical Workplace Learning NAQ-R scale items

**Figure 1:**
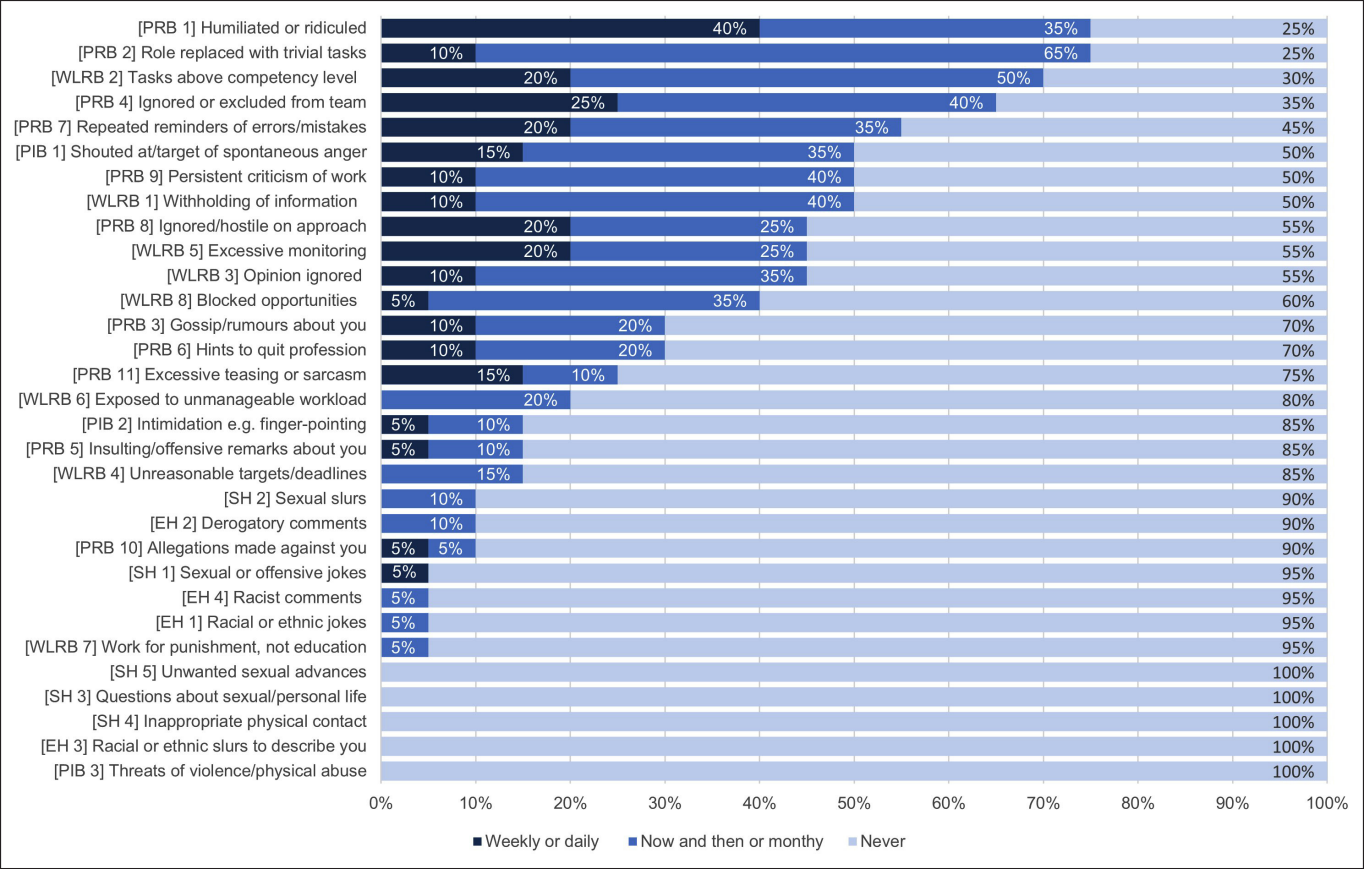
Percentage of respondents who experienced negative behaviours during clinical placement (n = 20). The CWL-NAQ-R items are categorised according to the type of negative act and item number. Categories include WLRB = Work Learning-Related Bullying, PRB = Person-Related Bullying, PIB = Physical Intimidation Bullying, EH = Ethnic Harassment, and SH = Sexual Harassment.

The negative experiences reported by participants were categorized into the five CWL NAQ-R themes, including (a) workplace learning-related bullying, (b) person-related bullying, (c) physically intimidating bullying, (d) ethnic harassment, and (e) sexual harassment. Almost all respondents (n = 19; 95%,) had experienced at least one negative behaviour during their final year of placement, 6 (30%) and 7 (35%) had experienced at least one negative behaviour daily or weekly, respectively. Participants reported person-related bullying as the most frequent issue (n = 19; 95%), followed by workplace learning-related bullying (n = 17; 85%). Table 1 provides a summary of the CWL NAR-R results and Figure 1 displays the frequency of the individual negative acts.

**Table 1:** Summary of results of the CWL NAQ-R. The mean value represents the frequency of experiences reported on the 0–5 scale.


CATEGORIES OF NEGATIVE ACTS	MEAN (SD) FREQUENCY	PARTICIPANTS n (%)

Workplace learning-related bullying	1.63 (±1.47)	17 (85%)

Person-related bullying	1.82 (±1.75)	19 (95%)

Physically intimidating bullying	1.43 (±1.25)	10 (50%)

Ethnic harassment	1.05 (±0.35)	2 (10%)

Sexual harassment	1.05 (±0.37)	2 (10%)


### Part C: Bullying

Of the 20 participants, 10 (50%) reported that the incivility experienced could be classified as bullying based on the definition provided in the survey. Clinical staff emerged as the most common perpetrators of the bullying acts, with all 10 respondents indicating that a clinician or clinical educator was the perpetrator. This was followed by the clinic manager (n = 5; 50%). The bullying incidents were most frequently during the patient consultation (n = 5; 50%), with (n = 2; 20%) participants reporting that the acts occurred when no one else was present.

#### Self-reported consequences of bullying

**Figure 2:**
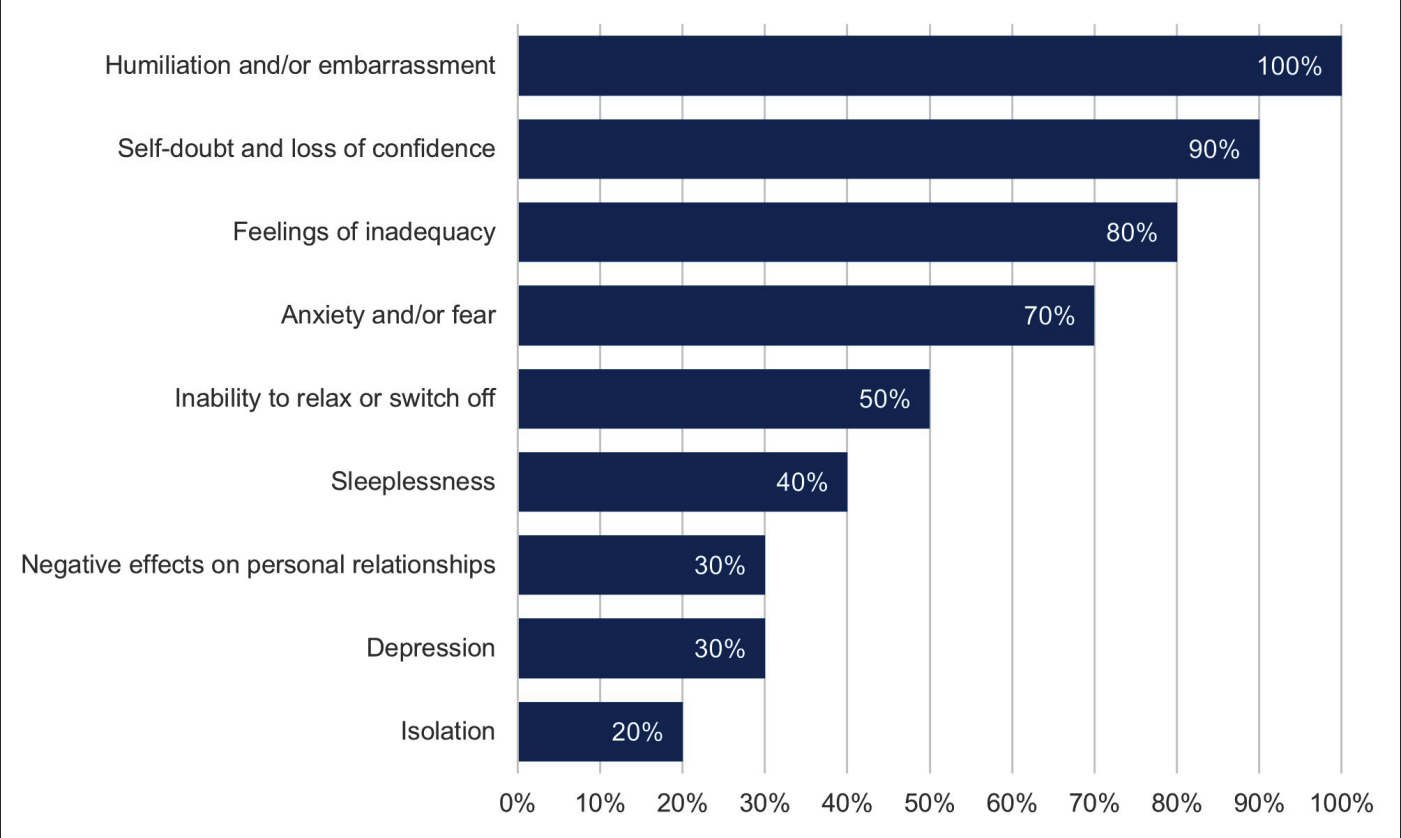
Self-reported consequences of bullying (n = 10).

Participants reported a range of consequences due to the bullying experienced, with the most common a sense of humiliation or embarrassment (n = 10; 100%), self-doubt or loss of confidence (n = 9; 90%), feelings of inadequacy (n = 8; 80%), and feelings of anxiety and fear (n = 7; 70%) (Figure 2). As a result of the bullying, a significant number of participants (n = 8; 80%) reported contemplated leaving their clinical placement, whilst (n = 5, 50%) considered leaving their course or intended career path.

#### Reporting of incidents

Of the 10 participants who reported experiencing bullying, almost all (n = 9; 90%) indicated that they did not report the behaviour. The reason for not reporting, included fear of the effect on reputation (n = 3; 30%), lack of knowledge on the reporting process (n = 3; 30%), and a preference for maintaining anonymity (n = 2; 20%). One of the respondents indicated they did not have the time to report, whilst one other reported that they focused on the fact that the placement would end.

The one respondent who did report bullying, reported the behaviour to clinic management but not the university. They reported that action was taken, but that they were not aware of the specifics of this action. They considered the handling of the incident as ‘very poor’, but details as to why the handling was considered poor were not reported.

## Discussion

This is the first study to investigate bullying of orthoptic students during clinical placements. Findings showed that that almost all students who responded had encountered at least one negative act during their placements, with 50% reporting that the behaviour was systematic and could be classified as bullying. Person-related bullying, or actions that target the individual, was the most common type of negative behaviour experienced, followed by workplace learning-related bullying, behaviour that targets the individual’s role, duties, or tasks. These experiences are consistent with previous research in allied health (Šarkić et al., in press; Stubbs and Soundy, 2013; Whiteside et al., 2014), which have shown high rates of bullying and harassment in this environment.

This study also demonstrated the negative consequences of bullying, with most orthoptic students reporting feelings of humiliation, self-doubt, and loss of confidence from the incidents and with some reporting sleep disturbances, depression, and affected personal relationships. The impact of being exposed to bullying and harassment on placement is well documented, particularly within the nursing literature (Birks et al., 2018; Budden et al., 2017; Clarke Colette et al., 2012; Minton and Birks, 2019; Ren et al., 2015). These studies have shown an association between the negative experiences on placement and psychological wellbeing, with many students on placement reconsidering their career path. This is consistent with our findings where 50% of orthoptic students who reported being bullied considered leaving the course and their intended career.

Unfortunately, 90% of orthoptic students who indicated they had been bullied did not report the behaviour experienced. Barriers varied from concerns regarding the effect on reputation and the desire for anonymity to being unaware of how to report bullying. Given that the perpetrators of the bullying were primarily clinical staff, it is likely that orthoptic students felt vulnerable by reporting an incident. The desire for anonymity and reputational concerns suggests that there may be a fear a of retaliation. It is noteworthy, however, that many students also indicated that they were not familiar with the bullying policies of the university, and some were unaware of how to report incidents. This suggests there is a need to better educate students prior to placement. There is also an opportunity to integrate lessons regarding bullying within the curriculum as part of topics such as healthcare ethics (Hakojärvi et al., 2014). Indeed, research has shown that education and training against bullying can be successful in reducing incidents (Branch and Murray, 2015). Creating a more transparent reporting process that recognises the power dynamics is likely also important to ensure accountability among both perpetrators and those responsible for intervention.

### Limitations

This study had various limitations, notably a small sample size and self-selection bias. With only 20 participants, the broader generalisability of the findings is limited. Due to the small sample size, it was also not possible to explore correlations between bullying and vulnerable groups of students. Moreover, the prevalence of reported negative experiences may be over-estimated as there is the potential that those who encountered bullying were more likely to complete the survey to disclose their experiences.

Additionally, the study did not account for the number of clinicians or staff encounters students were exposed to during their placements, which likely differed depending on the placement setting. In particular, if encounters were limited by a small sample of staff, the study may not have fully captured the diversity of student experiences. Another limitation is the lack of information on the mental health of students prior to or during placements. The experiences of students with existing mental health challenges, as well as the impact of these experiences, may also be shaped by these factors, which were not captured in this study.

The structured nature of the survey also limits the ability to explore the lived experiences of individuals. The use of a survey with forced choice responses, while valuable for capturing a snapshot of bullying on placements, cannot fully capture the complex nature and contextual nuances of student experiences. Future research should include qualitative methods with interviews or focus groups to gain more in-depth insights into the experiences of students on placement.

## Conclusion

This study is the first to investigate the prevalence of bullying amongst undergraduate orthoptic students during their clinical placements. The findings indicate that orthoptic students are exposed to incivility, bullying, and harassment, and suggest that universities must better prepare students for their clinical placement experience, including familiarising them with the bullying and harassment policies and procedures. Healthcare facilities and the orthoptic community must also be cognisant of the experiences of students on placement and its impact on their wellbeing. Addressing the issue of bullying in orthoptics will likely require a multifaceted approach where initiatives include not only enhancing awareness of the issue, but also include developing comprehensive anti-bullying education programs with an aim to proactively prevent instances of bullying and to create a more supportive and inclusive environment within healthcare facilities.

## Additional File

The additional file for this article can be found as follows:

10.22599/bioj.368.s1 Appendix 1. Part A to C.
