# Supplementary material for: Bullying and Incivility Experiences of Undergraduate Orthoptic Students on Clinical Placement
Source: Br Ir Orthopt J. 2025 Mar 21;21(1):36–42. doi: 10.22599/bioj.368 (PMC11927677; doi:10.22599/bioj.368)
Supplement: Appendix 1. — Part A to C. [file bioj-21-1-368-s1.pdf]

Survey: Bullying and Incivility Experiences of Undergraduate Orthoptic Students on Placements

**Part A Demographic Information (and policy awareness)**	
1. Please describe your gender.	Female<br>Male<br>Transgender Female<br>Transgender Male<br>Gender Variant/Non-conforming<br>Prefer not to say<br>Other (Specify)
2. Are you of Aboriginal or Torres Strait Islander origin?	No<br>Yes, Aboriginal<br>Yes, Torres Strait Islander<br>Yes, both Aboriginal and Torres Strait Islander
3. Is your primary language (the language you are most proficient in) English?	Yes<br>No
4. Do you speak a language other than English at home?	No, English only<br>Yes, Arabic<br>Yes, Cantonese<br>Yes, Greek<br>Yes, Italian<br>Yes, Mandarin<br>Yes, Vietnamese<br>Yes, Other (Specify)
5. What is your age?	20-30<br>31-40<br>41-50<br>51-60<br>Other (Specify)
6. Which of the following best describes you?	Final year Bachelor / Masters student<br>First year graduate<br>Other (Specify)
7. Which of the following clinical placement(s) apply to you? (select all that apply)	Public Hospital Outpatient Clinic<br>Private Ophthalmology Practice<br>Low Vision / Rehabilitation Clinic<br>Other (Specify)
8. Which of the following regions did you attend clinical placement? (select all that apply)	Metropolitan<br>Regional<br>Remote<br>International<br>Other (Specify)
9. Have you been made aware of the bullying and harassment policy at your clinical placement?	No<br>Yes<br>Can't remember/Don't know<br>Other (Specify)
10. Have you been made aware of the bullying and harassment policy at your university as related to clinical placements?	No<br>Yes<br>Can't remember/Don't know<br>Other (Specify)
11. When you commenced your clinical placement, did you receive information and training explaining how to deal with bullying and harassment, if experienced?	No<br>Yes<br>Can't remember/Don't know<br>Other (Specify)

**Part B Clinical Workplace Learning NAQ-R Scale Items** (From Smith-Han et al., 2020)<br>Five response options for each scale (Never, Now and Then, Monthly, Weekly, Daily)	
Whilst on placement in final year, how often were you subjected to the following negative acts?<br>Please check the box that best corresponds with your experience.	
**Workplace Learning-Related Bullying (WLRB)**	
WLRB 1	Someone withholding information which affects your learning
WLRB 2	Being ordered to do tasks above your level of competence
WLRB 3	Having your opinions and views ignored
WLRB 4	Being given tasks with unreasonable or impossible targets or deadlines
WLRB 5	Excessive monitoring of your work
WLRB 6	Being exposed to an unmanageable workload
WLRB 7	Being assigned work for punishment rather than for educational value
WLRB 8	Having learning opportunities blocked or withheld by Other (Specify)s
**Person-Related Bullying (PRB)**	
PRB 1	Being humiliated or ridiculed in connection with your learning
PRB 2	Having key areas of your student role removed or replaced with more trivial or unpleasant tasks
PRB 3	Spreading of gossip and rumours about you
PRB 4	Being ignored or excluded from the clinical team
PRB 5	Having insulting or offensive remarks made about your person (i.e. habits and background), your attitudes or your private life
PRB 6	Hints or signals from Other (Specify)s that you should quit studying your profession
PRB 7	Repeated reminders of your errors or mistakes
PRB 8	Being ignored or facing a hostile reaction when you approach
PRB 9	Persistent criticism of your work and effort
PRB 10	Having allegations made against you
PRB 11	Being the subject of excessive teasing and sarcasm
**Physically Intimidating Bullying (PIB)**	
PIB 1	Being shouted at or being the target of spontaneous anger
PIB 2	Intimidating behaviour such as finger-pointing, invasion of personal space, shoving, blocking/barring the way
PIB 3	Threats of violence of physical abuse or actual abuse
**Sexual Harassment (SH)**	
SH 1	Sexually explicit or offensive jokes
SH 2	Sexual slurs
SH 3	Questions or insinuations about your sexual or private life
SH 4	Inappropriate physical contact
SH 5	Unwanted sexual advances
**Ethnic Harassment (EH)**	
EH 1	Told jokes about your racial or ethnic group
EH 2	Made derogatory comments about your racial or ethnic group
EH 3	Used racial or ethnic slurs to describe you
EH 4	Made racist comments (for example, says people of your ethnicity aren’t very smart or can’t do the job)

Part C Experience of Bullying	
Consider your responses in **Part B** and the definition of bullying below.<br><br>"Bullying takes place when one or more persons systematically and over time feel that they have been subjected to negative treatment on the part of one or more persons, in a situation in which the person(s) exposed to the treatment have difficulty in defending themselves against them. It is not bullying when two equally strong opponents are in conflict with each Other (Specify)" (Einarsen, Raknes, Matthiesen, & Hellesøy, 1994, p. 20)	
1. Given the above definition, do you feel that you have been subjected to bullying during your clinical placement?	No<br>Yes
**If yes:**	
2. When did the incident(s) occur?	During patient consultation<br>Prior to patient consultation<br>During a break<br>When no one was around<br>Other (Specify)
3. Who was the perpetrator of the bullying? (select all that apply)	Clinical Educator<br>Clinical Staff (i.e. medical staff, nursing staff, orthoptist)<br>Clinic Manager<br>Administrative staff<br>Patient<br>Students<br>Other (Specify)
4. Which of the below have you experienced due to bullying or harassment? (select all that apply)	Anxiety/fear<br>Depression<br>Feelings of inadequacy<br>Humiliation/embarrassment<br>Inability to relax or switch off from work or study<br>Isolation<br>Self-doubt and loss of confidence<br>Sleeplessness<br>Negative effects on personal relationships<br>Other (Specify)
5. Have you ever reported the incident(s) of bullying?	No<br>Yes<br>Other (Specify)
5a. If no, which of the following would explain why?	Concern about your reputation<br>Desire not to have my name on an incident report<br>Fear of not gaining employment<br>Lack of support from the university<br>I did not know where/how to report it<br>Too busy<br>Other (Specify)
5b. If yes, who did you report the incident to?	University staff member<br>Clinic management<br>Police<br>Other (Specify)
5c. Was action taken in response to your report?	No<br>Yes<br>Not sure
5d. If yes, what action was taken after you reported the incident?	No action<br>Disciplinary action against the perpetrator by their employer<br>Disciplinary action against the perpetrator by my university<br>Not sure<br>Other (Specify)

5e. What are your thoughts on the way the incident was handled?	Excellent<br>Good<br>Satisfactory<br>Poor<br>Very poor
6. Have you thought about leaving your clinical placement due to the bullying?	No<br>Yes<br>Other (Specify)
7. Have you ever thought about leaving your course due to the bullying?	No<br>Yes<br>Other (Specify)
